# Bioconversion of waste glycerol into viscosinamide by *Pseudomonas fluorescens* DR54 and its activity evaluation

**DOI:** 10.1038/s41598-024-51179-4

**Published:** 2024-01-17

**Authors:** Dominika Jama, Wojciech Łaba, Mateusz Kruszelnicki, Izabela Polowczyk, Zbigniew Lazar, Tomasz Janek

**Affiliations:** 1 Department of Biotechnology and Food Microbiology, Wrocław University of Environmental and Life Sciences, 51-630 Wrocław, Poland; 2 Department of Process Engineering and Technology of Polymers and Carbon Materials, Wroclaw University of Science and Technology, 50-370 Wrocław, Poland

**Keywords:** Biotechnology, Industrial microbiology

## Abstract

Lipopeptides, derived from microorganisms, are promising surface-active compounds known as biosurfactants. However, the high production costs of biosurfactants, associated with expensive culture media and purification processes, limit widespread industrial application. To enhance the sustainability of biosurfactant production, researchers have explored cost-effective substrates. In this study, crude glycerol was evaluated as a promising and economical carbon source in viscosinamide production by *Pseudomonas fluorescens* DR54. Optimization studies using the Box − Behnken design and response surface methodology were performed. Optimal conditions for viscosinamide production including glycerol 70.8 g/L, leucine 2.7 g/L, phosphate 3.7 g/L, and urea 9.3 g/L were identified. Yield of viscosinamide production, performed under optimal conditions, reached 7.18 ± 0.17 g/L. Preliminary characterization of viscosinamide involved the measurement of surface tension. The critical micelle concentration of lipopeptide was determined to be 5 mg/L. Furthermore, the interactions between the viscosinamide and lipase from *Candida rugosa* (CRL) were investigated by evaluating the impact of viscosinamide on lipase activity and measuring circular dichroism. It was observed that the α-helicity of CRL increases with increasing viscosinamide concentration, while the random coil structure decreases.

## Introduction

The interest in biosurfactants has increased over the past decades due to their low toxicity, high biodegradability, and effectiveness under extremes of temperature, pH, and salinity, compared to synthetic surfactants^1^. These compounds are surface-active biomolecules containing both hydrophobic and hydrophilic domains^2^. Several microorganisms including bacteria, fungi, and yeast produce these amphiphilic molecules^3^. Biosurfactants are capable of reducing surface tension (ST) and interfacial tension, and have properties such as emulsification, foaming, and wetting^4,5^. Consequently, biosurfactants have a wide range of applications in various industries including pharmaceutics, cosmetics, agriculture, and food production^6–8^. Additionally, several biosurfactants exhibit anticancer, antibacterial, and antifungal activities^9,10^. They are also capable of inhibiting the adhesion of pathogenic microorganisms to various surfaces^11^.

Biosurfactants can be classified into various groups, including lipopeptides, glycolipids, phospholipids, fatty acids, and polymeric surfactants, based on their chemical structures^2^. Among them, lipopeptides are the most popular and intriguing class of microbial surfactants. Viscosinamide, a cyclic lipopeptide belonging to the viscosin group, is produced by non-ribosomal peptide synthetases in the *Pseudomonas* species^12^. The amino acid sequence of this secondary metabolite is L-Leu-D-Gln-D-aThr-D-Val-L-Leu-D-Ser-L-Leu-D-Ser-L-Ile. Viscosinamide is cyclized via an ester bond between the C-terminal carboxylic acid and the side-chain hydroxyl moiety of Thr3^12,13^.

The manufacturing process of synthetic surfactants involves utilizing a variety of chemical substances, which are commonly obtained from petroleum or other petrochemical raw materials and are known for their toxicity and environmental effects^2^. As a result, microbial surfactants have garnered increased attention due to their ability to be synthesized from low-cost agro-industrial wastes, offering economical, renewable, and eco-friendly substrates^14^. However, the utilization of waste materials for the production of lipopeptide biosurfactants is not an ideal solution due to the limitations, including low process efficiency. The concentrations of another lipopeptide, surfactin, typically range from 0.001 to 3 g/L using various types of wastes, underscoring the challenge of achieving optimal yields in the production process^15,16^. In this study, the production process of a lipopeptide biosurfactant, viscosinamide, was conducted utilizing raw glycerol, which is a biodegradable, cheap, and industrial carbon source primarily generated from biodiesel, soap, or stearin production. In addition to selecting relevant carbon sources, optimizing the media composition has a significant influence on biosurfactant production, which was also investigated in this study^1^.

The activity of lipases can be enhanced by the presence of biosurfactants^17^. Several studies have described the interactions between lipases and synthetic or microbial surfactants^17–19^. Lipases (triacylglycerol hydrolases, EC 3.1.1.3) catalyze the hydrolysis of triacylglycerols to glycerol and long-chain fatty acids^20^. These enzymes play essential roles as biocatalysts for biotechnological applications such as in detergent, dairy, food, fat and oil, cosmetics, and pharmaceutical industries^21,22^. Lipases appear to offer an environmentally friendly alternative to decreasing the usage of commonly employed chemicals^23^. However, further research is on the interactions between biosurfactant–lipase systems.

Experimental designs based on response surface methodology (RSM) are a group of statistical and mathematical tools that provide a detailed plan for collecting data and using them to identify causal relationships between variables. These designs are based on multiple regression analysis, employing a quadratic function to describe the impact of input variables on the output variable (response). RSM is particularly useful in modeling non-linear dependencies between multiple variables, as it reduces the number of experiments that need to be performed, maximizing the amount of information derived from experiments, as well as provides controlled conditions for systematic data collection. Moreover, RSM designs allow the interactions between variables to be defined, which are overlooked in the standard one-variable-at-a-time (OVAT) approach. The methodology comprises consecutive steps: selection of variables that significantly affect the response, model identification and evaluation supported by residual analysis, and response optimization^24,25^.

The aim of this study was to evaluate the production of viscosinamide by *P. fluorescens* DR54 using low-cost culture media, formulated using waste glycerol from biodiesel, stearin, and soap production processes. Additionally, the effects of viscosinamide on the conformation and activity of lipase from *Candida rugosa* (CRL) were evaluated using circular dichroism (CD), ST, and the enzyme activity assay.

## Results and discussion

### Growth of *P*. *fluorescens* DR54 on crude glycerol

**Figure 1:**
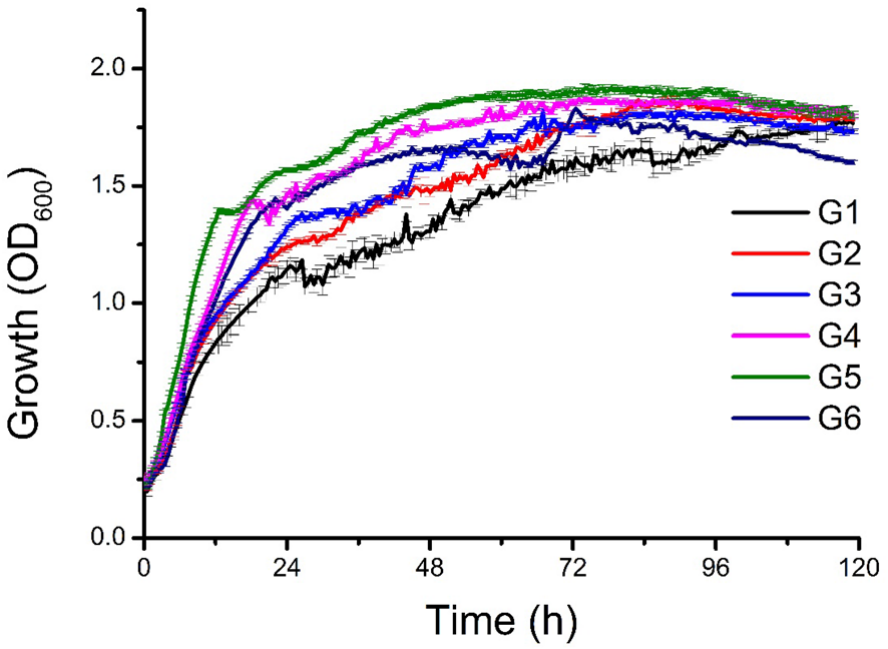
Growth curves of *P. fluorescens* DR54 growing on MSM supplemented with pure (G1) and crude glycerol (G2–G6). The experiments were performed at 28 °C under constant agitation (180 rpm) using a microplate reader Spark Tecan in triplicate for each type of glycerol.

Biosurfactants are derived from many substrates including agricultural, food, and industrial wastes^14^. Importantly, the utilization of low-cost substrates not only reduces production costs but also allows the valorization of byproducts. The selection of a carbon source significantly impacts lipopeptide biosurfactant production^1^. In this study, the effect of waste glycerol on *P. fluorescens* DR54 growth was investigated in mineral salt medium (MSM) containing various samples of crude glycerol. Pure glycerol was used as a reference experiment. As seen in Fig. 1, the increased growth of *P. fluorescens* DR54 was observed in media containing crude glycerol (G2–G6) compared to pure glycerol (G1). The batch of waste glycerol derived from soap production (G5) showed the shortest exponential phase. In this sample, the highest growth profile and final optical density measurements at 600 nm (OD_600_) were also observed. That experiment showed that *P. fluorescens* DR54 has the ability to be effectively utilized for growth various batches of crude glycerol derived from industry.

### Evaluation of crude glycerol from industrial wastes as a substrate for biosurfactant production during the shake flask experiment

**Figure 2:**
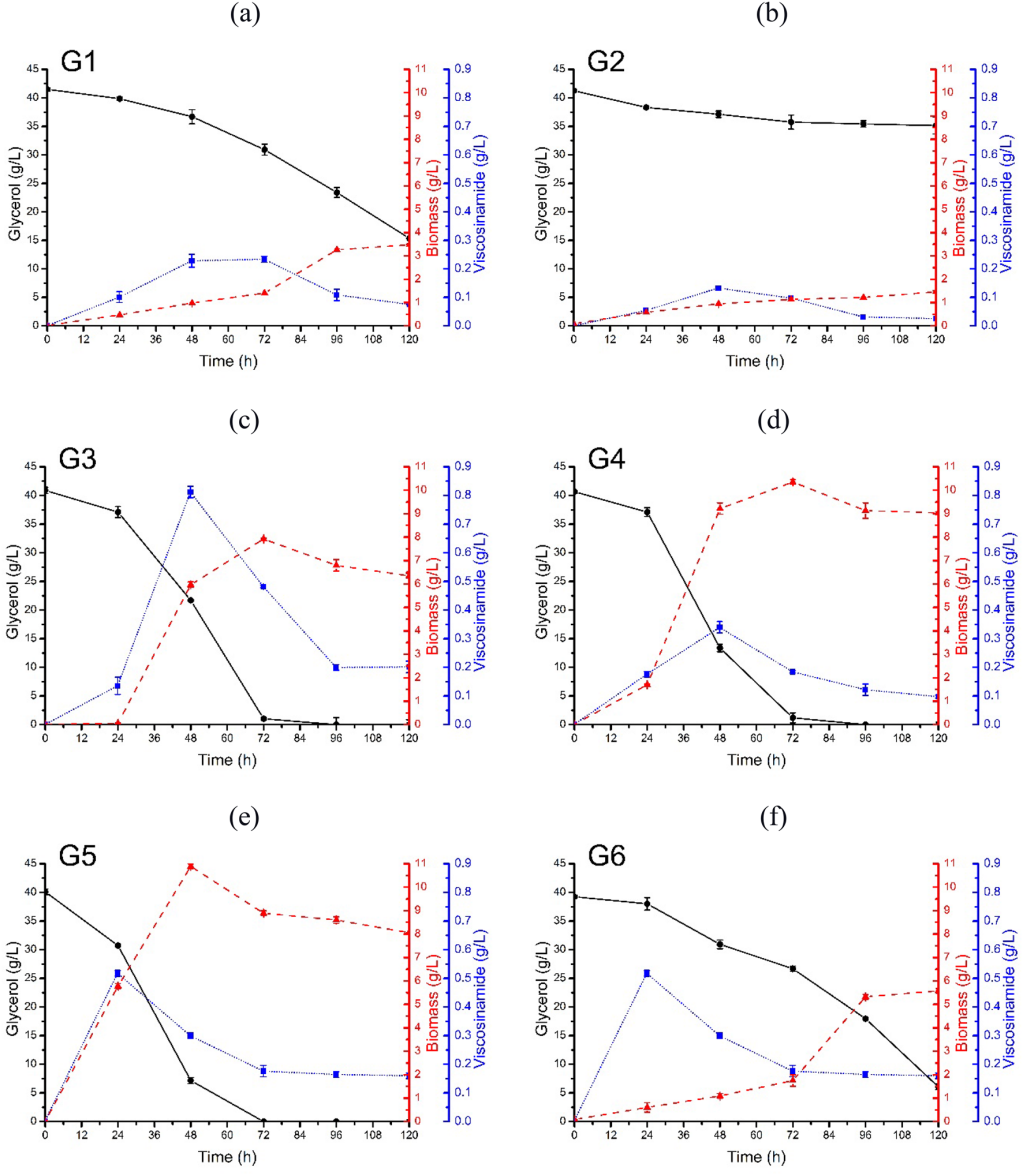
Glycerol consumption (g/L), growth of *P. fluorescens* DR54, and viscosinamide production (g/L) performed in MSM supplemented with 40 g/L pure (G1) and waste glycerol (G2–G6) from diverse sources. Experiments were performed at 28 °C and 180 rpm for 120 h.

The application of low-cost carbon sources is an attractive alternative for lipopeptide biosurfactant production by microorganisms. Phulpoto and coworkers observed that glycerol/urea is one of the optimal carbon/nitrogen sources for biosurfactant production by *Pseudomonas* sp. S2WE^26^. Other studies comparing different carbon sources have shown that glycerol is one of the most favorable substrates for biosurfactant production^2,27^. Thus, in the next step, the possibility of biosurfactant biosynthesis was analyzed during the shake flask experiment (Fig. 2a–f). Depending on the substrate used, diversity in viscosinamide concentration or biomass concentration could be observed. The highest viscosinamide concentration 0.8 g/L (Fig. 2c) was noted at 48 h of culture in a medium containing glycerol derived from biodiesel production (G3). The concentration of lipopeptide biosurfactant reported in this case was almost 4 times higher than the concentration observed in the medium with pure glycerol (0.2 g/L; Fig. 2a). In another study, the concentration of the lipopeptide biosurfactant, derived from crude glycerol obtained from the biodiesel industry as a carbon source, reached 158 mg/L after 72 h^28^. The fastest biosurfactant biosynthesis was noted in 24 h of growth in a medium containing glycerol delivered from soap production (G5) (Fig. 2e), while the last batch viscosinamide produced was reached at 72 h when G6 glycerol (Fig. 2f) was used as a carbon source. Stearin-derived waste glycerol (G2) (Fig. 2b) seems to be the least favorable source of carbon for biosurfactant production. This was the only case when the viscosinamide concentration (0.1 g/L) was lower than that obtained in the control experiment. This culture was also characterized by a lower biomass concentration. In the studies, waste glycerols with varying composition and impurity levels were utilized (Tables S1 and S2). Presumably, the observed differences in viscosinamide production using different samples of waste glycerol can be attributed to their distinct compositions. A similar situation has been previously described in the context of surfactin production^16^.

### Optimization of the culture medium for viscosinamide production by *P*. *fluorescens* DR54

Assays of crude glycerol from industrial waste as a substrate during the shake flask experiment allowed us to choose the most advantageous carbon source for viscosinamide production. To achieve the highest concentration of biosurfactant the optimization of the culture medium was conducted. The regression model obtained from the Box–Behnken experimental design allowed us to establish the collective impact of four components of the culture medium.

**Figure 3:**
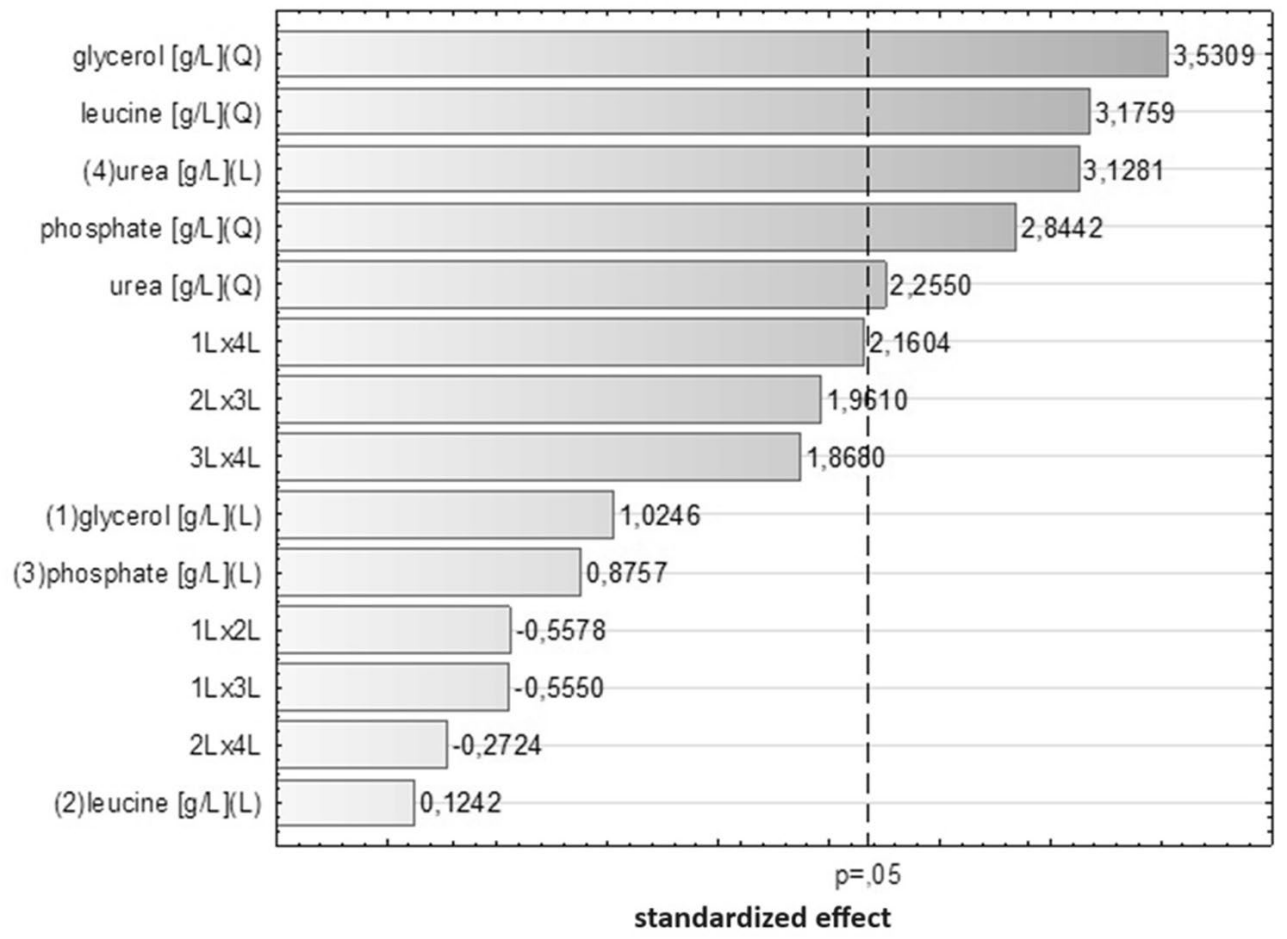
Pareto chart of standardized effects.

According to the Pareto chart, all independent variables exerted a statistically significant impact on the response (Fig. 3, Table 1). The strongest influence on viscosinamide production was bound to the concentration of the main substrate (i.e. glycerol), followed by equally significant leucine and urea. The lowest influence observed was that of phosphate. The observed relationships were predominantly non-linear, as linear regression coefficients were confirmed to be statistically insignificant, with the exception of the linear coefficient of urea. In addition, no significant interaction between independent variables was observed.

**Table 1:** Summary of effects of the regression model.

Variable	Coefficient	Standard error	t-value	p-value
Intercept	0.0197	0.6264	0.0315	0.9754
X_1_	1.1117	1.0850	1.0246	0.3258
X_2_	0.1348	1.0850	0.1242	0.9032
X_3_	0.9502	1.0850	0.8757	0.3984
X_4_	3.3941	1.0850	3.1281	0.0087
X_1_X_1_	2.8733	0.8138	3.5309	0.0041
X_2_X_2_	2.5845	0.8138	3.1759	0.0080
X_3_X_3_	2.3145	0.8138	2.8442	0.0148
X_4_X_4_	1.8350	0.8138	2.2550	0.0436
X_1_X_2_	− 1.0483	1.8793	− 0.5578	0.5872
X_1_X_3_	− 1.0431	1.8793	− 0.5550	0.5891
X_1_X_4_	4.0602	1.8793	2.1604	0.0517
X_2_X_3_	3.6854	1.8793	1.9610	0.0735
X_2_X_4_	− 0.5119	1.8793	− 0.2724	0.7900
X_3_X_4_	3.5107	1.8793	1.8681	0.0864

X_1_:glycerol, X_2_: leucine, X_3_: phosphate, X_4_: urea.

Viscosinamide contains three molecules of leucine in the structure. The strong pronunciation of leucine found on the Pareto chart confirms our hypothesis that addition of this amino acid to culture medium as the precursor plays a crucial role in biosurfactant production. A similar correlation was observed in studies examining the production of pseudofactin by *P. fluorescens* BD5^29^. These studies demonstrated that amino acid supplementation is a helpful factor impacting lipopeptide production. The literature also includes descriptions of studies investigating the positive or negative influence of amino acids on surfactin production by various strains of *Bacillus* bacteria^30,31^.

Analysis of variance (ANOVA) was used to verify the significance of all effects included in the regression model. The coefficient of determination, R^2^ = 0.7821, indicated sufficient competence of the developed model (Table 2). The model with the R^2^ ≥ 0.6 (60%) can be considered as a valid model^32^.

**Table 2:** Analysis of variance (ANOVA) of the regression model, involving combined linear (L) and quadratic (Q) effects (L + Q) of input variables and interaction terms (X_a_*X_b_).

Regression model component	SS	df	MS	F	p
X_1_: glycerol [g/L] L + Q	47.739	2	23.870	6.758	0.0108
X_2_: leucine [g/L] L + Q	35.678	2	17.839	5.051	0.0256
X_3_: phosphate [g/L] L + Q	31.279	2	15.640	4.428	0.0363
X_4_: urea [g/L] L + Q	52.520	2	26.260	7.435	0.0079
Interactions X_1_*X_2_ X_1_*X_3_ X_1_*X_4_ X_2_*X_3_ X_2_*X_4_ X_3_*X_4_	1.099	1	1.099	0.311	0.5872
	1.0881	1	1.088	0.308	0.5811
	16.485	1	16.485	4.667	0.0517
	13.582	1	13.582	3.846	0.0735
	0.262	1	0.262	0.074	0.7900
	12.325	1	12.325	3.490	0.0864
Residual error	42.382	12	3.532		
Total SS	194.465	26			

R^2^ = 0.7821.

The calculated regression coefficients allowed us to define the following polynomial equation (significant terms underlined) (1):

1 $${\text{Y}} = \, - {4}.0{43 } + \, \underline{0.{14}0{\text{X}}_{{1}}} + { 1}.{\text{693X}}_{{2}} + { 1}.{2}0{\text{3X}}_{{3}} + \, 0.{4}0{\text{5X}}_{{4}} - \, \underline{0.00{\text{1X}}_{{1}} {\text{X}}_{{1}}} - \, \underline{0.{\text{431X}}_{{2}} {\text{X}}_{{2}}} - \, \underline{0.{\text{457X}}_{{3}} {\text{X}}_{{3}}} - \, \underline{0.0{9}0{\text{X}}_{{4}} {\text{X}}_{{4}}} - \, 0.00{\text{5X}}_{{1}} {\text{X}}_{{2}} - \, 0.00{\text{5X}}_{{1}} {\text{X}}_{{3}} + \, 0.0{1}0{\text{X}}_{{1}} {\text{X}}_{{4}} + \, 0.{\text{334X}}_{{2}} {\text{X}}_{{3}} - \, 0.0{\text{23X}}_{{2}} {\text{X}}_{{4}} + \, 0.{\text{173X}}_{{3}} {\text{X}}_{{4}} ,$$

**Figure 4:**
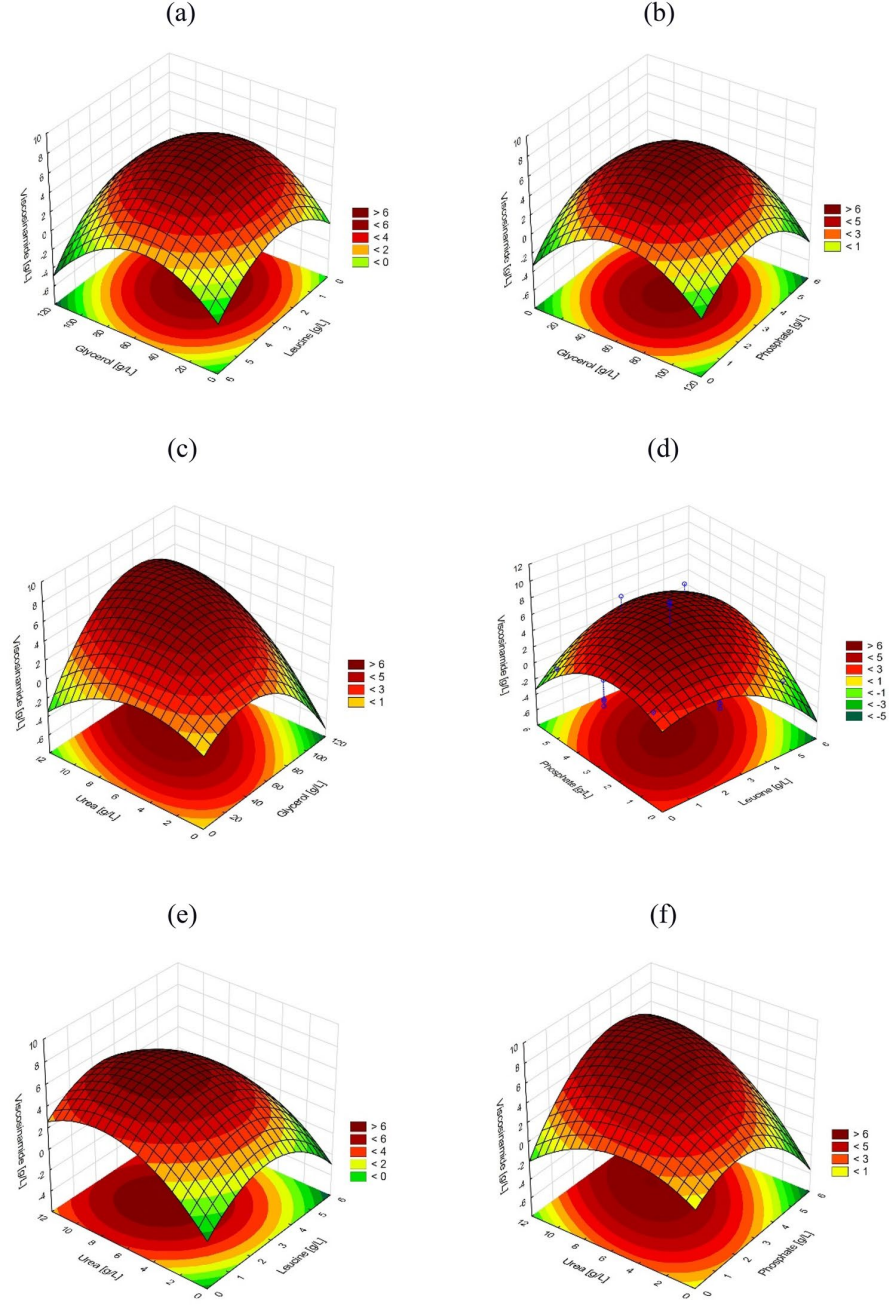
Response surface analysis. Effects of (**a**) glycerol and leucine, (**b**) glycerol and phosphate, (**c**) urea and glycerol, (**d**) phosphate and leucine, (**e**) urea and leucine, and (**f**) urea and phosphate on the concentration of viscosinamide.

To analyze the combined effects of the four factors on the viscosinamide production, response surface plots were prepared that represent the graphical form of the regression equation. The surface plots that illustrate the shared effects of pairs of independent variables exhibited a distinctive convex curvature, which allowed us to establish clear optima to maximize the response. The influence of the main substrate’s concentration of viscosinamide production exhibited a nearly symmetrical curvature with its maximum to the center of the experimental layout (Fig. 4a,b). Comparable symmetry was observed in the case of plots for leucine (Fig. 4a,d,e), phosphate (Fig. 4b,f), and glycerol (Fig. 4c). For the concentration of urea, however, maximal viscosinamide output occurred in a higher concentration, far above the central point of 6 g/L.

Response optimization of the regression model provided for the following optima for independent variables: glycerol 70.8 g/L, leucine 2.7 g/L, phosphate 3.7 g/L, and urea 9.3 g/L, to yield a predicted response of 7.34 g/L viscosinamide with a confidence interval of 5.2–9.5 g/L. Moreover, when the process was conducted under optimal conditions, the amount of viscosinamide produced reached 7.18 ± 0.17 g/L, which validated the model predictions. The result was almost nine times higher compared to the concentration of viscosin obtained before the optimization process (Fig. 2c).

RSM methodologies have been used for a multitude of successful scientific applications, including designing culture media composition and cultivation parameters for various biotechnological processes, for example, the biosynthesis of antimicrobial di-(2-ethylhexyl) phthalate (DEHP) by *Bacillus subtilis*^33^. Here, a combination of OVAT methodology, Plackett–Burman design for the screening of influential variables, and Box–Behnken design for the non-linear modeling of three independent variables allowed us to successfully establish optimal culture conditions that translated into nearly two-fold DEHP activity. Likewise, production of a Parkinson’s disease treatment compound 3,4-dihydroxyphenyl-L-alanine by *Pseudomonas* sp. SSA underwent a similar optimization procedure^34^. The authors used a four-variable Box–Behnken design for modeling the simultaneous impacts of glucose, peptone, tyrosine, and copper sulfate (CuSO_4_) on the production level of DOPA, followed by a successful optimization step to determine the desired levels of input variables to maximize the response.

Several studies have indicated that the production of biosurfactants can be influenced by the concentration of carbon substrates, nitrogen, or phosphate^4,35,36^. Ghribi and Ellouze-Chaabouni observed the highest concentration of lipopeptide biosurfactants (0.75 g/L) using 40 g/L glucose and 5 g/L urea as carbon and nitrogen sources, respectively^37^. In other studies, after process optimization, utilizing glycerol as a carbon source and amino acid supplementation, the concentration of pseudofactin reached 1.2 g/L^29^. On the other hand, Liu et al.^28^ by optimizing the fermentation process for surfactin production, obtained 2 g/L biosurfactant by using a medium containing sucrose and supplemented with ornithine. Furthermore, the amount of biosurfactants produced in media containing glycerol as a substrate usually ranges from 0.15 to 2.9 g/L^16,26,28,38–40^. To the best of our knowledge, no study has been conducted focusing on optimization of the viscosinamide production process.

### Surface activity determination

**Figure 5:**
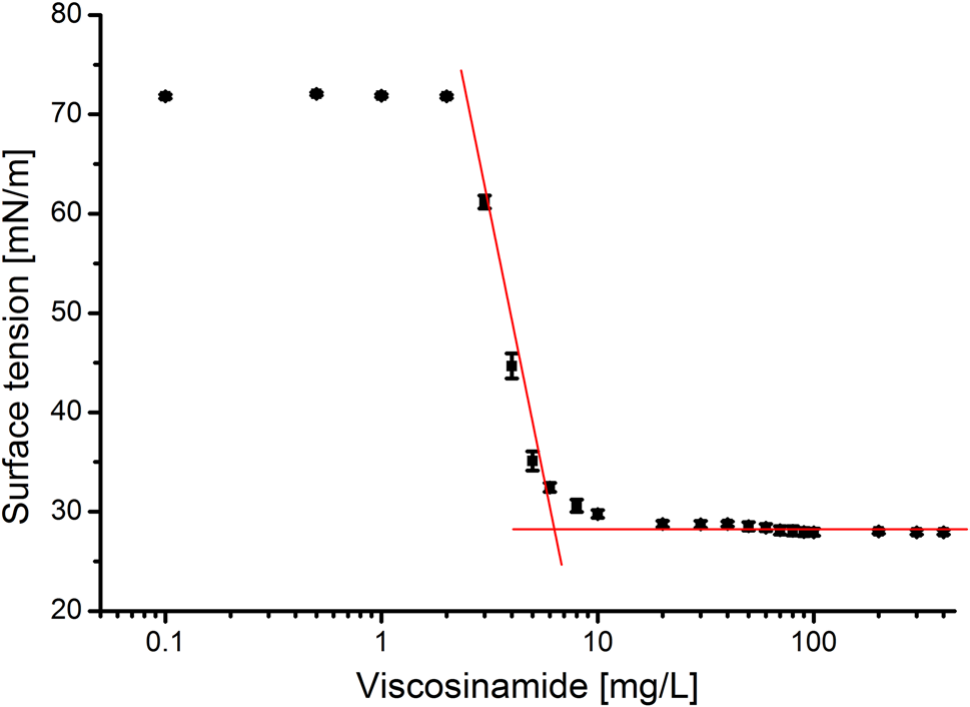
Effect of pure viscosinamide concentration (obtained at the optimal conditions) on surface tension. The critical micelle concentration (CMC) was determined from the intersection of regression lines that describe two parts of the curve, below and above the CMC.

ST and critical micelle concentration (CMC) are two important parameters characterizing biosurfactants. The ST of viscosinamide produced by *P. fluorescens* DR54 using waste glycerol derived from biodiesel production as a carbon source was determined using the pendant drop method. The lowest ST was 27.9 mN/m. The CMC value was determined from the ST versus viscosinamide concentration plot and was determined to be 5 mg/L (Fig. 5). Such a low value of CMC indicates that viscosinamide exhibits strong surface active properties. There are several studies measuring the surface activity of viscosinamide. Saini et al.^41^ observed CMC 54 mg/L for a ST of 27.5 mN/m. Renard et al.^42^ found a CMC value of 21.6 mg/L for a ST of 25 mN/m. However, in other studies, CMC of viscosinamide 0.15 mg/L and even 4 mg/L were measured for ST 26.5 and 25 mN/m, respectively^43,44^. Such differences may be due to differences in the purity of the analyzed lipopeptides. The results obtained from this study as well as previously published data indicate that viscosinamide is a powerful lipopeptide from the perspective of detergents, pharmaceuticals, cosmetics, and food application.

### Effect of viscosinamide on lipase activity

Most lipases have a hydrophobic region in the vicinity of the active site, which becomes exposed upon interaction with surfactants^45,46^. The exposure of this hydrophobic area enhances lipase activity against the substrates. The addition of surfactants such as sodium dodecyl sulfate and sodium dodecyl benzene sulfonate can induce a conformational change in CRL from a closed to an open form, leading to an increase in catalytic activity. This increase in activity is referring to the surfactant molecules disrupting the intermolecular interactions that maintain the lipase in the closed conformation, resulting in exposure of the hydrophobic region and hence, an increase in catalytic efficiency^46^. The effect of viscosinamide produced by *P. fluorescens* on the activity of CRL compared to the control sample is shown in Table 3.

**Table 3:** Effect of viscosinamide on CRL activity.

	Concentration (mg/L)	Relative activity (%)
Control		100 ± 0.5
Viscosinamide	5	112 ± 0.4
	10	124 ± 2.1
	20	145 ± 0.8
	40	182 ± 1.7
	80	193 ± 0.2
	160	171 ± 2.4

The results represent the average of triplicate experiments ± standard deviation (SD).

To determine the effect of biosurfactant on CRL activity, the baseline was established by setting the relative activity of CRL as 100% in the absence of biosurfactants, Interestingly, the presence of only 5 mg/L viscosinamide leads to an increase in CRL activity. The results showed that CRL activity was enhanced almost two-fold after adding 80 mg/L lipopeptide biosurfactant. A significant increase in CRL activity (82% compared to control) was also observed upon addition of 40 mg/L viscosinamide. Janek et al.^17^ showed that viscosinamide and amphisin have effects on the activity of lipase from *Yarrowia lipolytica*, suggesting that lipopeptide biosurfactants, as natural surfactants, have the ability to open the conformation of lipases. As a result, it is believed that viscosinamide creates an expedient environment for the substrate to link and interact with the active site of the lipase.

### CD measurement

**Figure 6:**
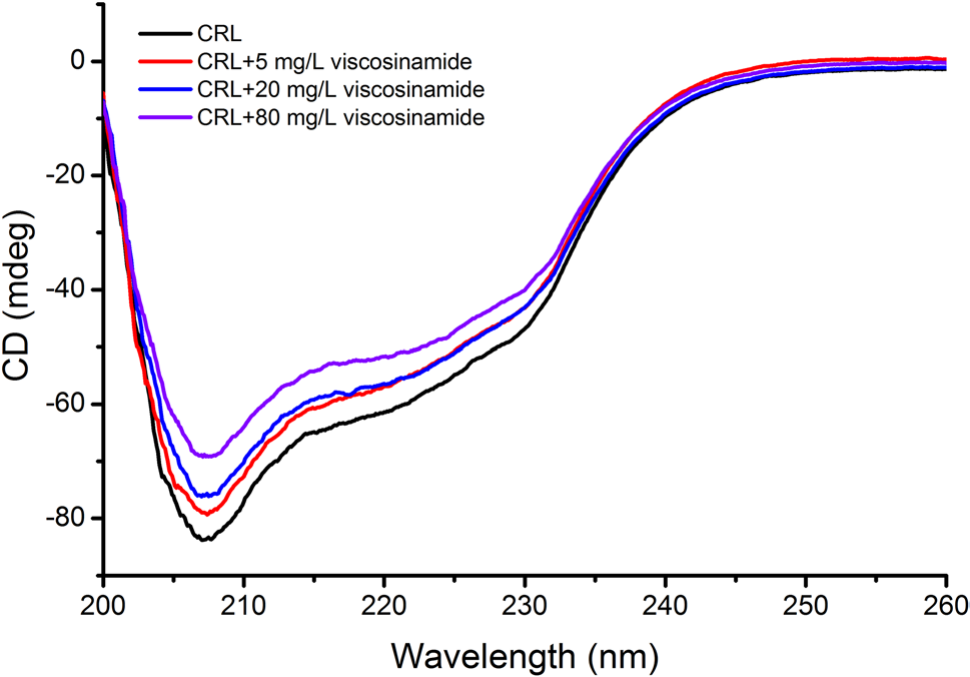
Circular dichroism (CD) spectra of lipase from *Candida rugosa* (1 g/L) alone and in the presence of different concentrations of viscosinamide.

Lipases are amphiphilic and the association with other amphiphilic substances, such as biosurfactants, is expected to occur^47^. This binding is complex and depends strongly on many different aspects, such as the nature of both species and of the solvent, concentrations, etc. CD is a crucial method that allows for the characterization of proteins and nucleic acids. CD spectroscopy is highly sensitive and enables the monitoring of even subtle changes in the tertiary and secondary structures of a protein when it interacts with drugs^48,49^. In this study, CD was used to determine the effect of viscosinamide addition on the secondary structure of lipase derived from *C. rugosa*. The CD spectrum of CRL shows negative bands at 208 and 222 nm (Fig. 6), which is specific of a high α-helical content^50^. Upon the addition of viscosinamide, the negative values at both minima decreased with increasing biosurfactant concentration, indicating a conformational change induced by the overall structure of viscosinamide and further formation of the CRL–viscosinamide complex. The results showed that upon interaction of CRL with viscosinamide, the α-helical content of CRL increases (Table 4). The α-helical content increased from 14.8% (when untreated) to 18.9% at a viscosinamide concentration of 80 mg/L. As the concentration of viscosinamide increased, an increase of α-helix, β-sheet, and β-turn content of CRL was also observed (Table 4). On the other hand, the random structure decreased. Similar dependencies were observed in studies of the mannosylerythritol lipids-A/β-glucosidase system using concentrations of 10 and 50 μM mannosylerythritol lipids-A^51^. The opposite trend was observed by Janek et al.^52^ in a bovine serum albumin/pseudofactin II system. Likewise, Gull and co-workers^53^ observed that the α-helical content significantly decreased with increases in cetyltrimethylammonium bromide concentration.

**Table 4:** Secondary structures of enzymes estimated from CD spectra.

	α-helix (%)	β-sheet (%)	β-turn (%)	Random (%)
CRL	14.8	21.5	13.5	50.2
CRL + 5 mg/L viscosinamide	15.4	22.6	13.9	48.1
CRL + 20 mg/L viscosinamide	16.1	23.4	14.7	45.8
CRL + 80 mg/L viscosinamide	18.9	26.3	18.4	36.4

## Conclusions

The identification of essential culture parameters is crucial for lipopeptide production, and achieving high concentrations can lead to a decrease in the production costs associated with these compounds. The results of this study showed that *P. fluorescens* DR54 can grow in MSM supplemented with waste glycerol obtained from various industrial processes, serving as a carbon source. Moreover, when the process was conducted under optimal conditions, the amount of viscosinamide produced reached 7.18 ± 0.17 g/L, which validated the model predictions (7.34 g/L viscosinamide, with a confidence interval of 5.2–9.5 g/L). The preliminary characterization of the biosurfactant revealed its capability to reduce ST. The CMC value of ST was determined to be 5 mg/L with a ST of 27.9 mN/m. Additionally, interactions occurring in the viscosinamide–lipase system were investigated. It was observed that the presence of biosurfactant led to an increase in CRL activity and influenced its structure. The results from this study could be useful for developing the applications of lipopeptide and CRL in many industries including detergents, pharmaceuticals, cosmetics, and food production.

## Materials and methods

### Chemicals and reagents

All chemicals and reagents were of analytical or liquid chromatography (LC)–mass spectrometry grade, purchased from Merck Co. (Darmstadt, Germany). Five different batches of crude glycerol from bio-diesel (G3, G4, G6), stearin (G2), and soap (G5) were delivered from companies located in Poland (Grupa Azoty, Orlen, and Lotos.) The composition of the five crude glycerol samples was previously reported^54^ and is listed in Tables S1 and S2. Pure glycerol was obtained from POCH SA (Gliwice, Poland).

### Microorganisms

*P. fluorescens* DR54^55^ used in this study for viscosinamide production was graciously provided by Dr. Ole Nybroe (University of Copenhagen, København, Denmark) and stored at the Department of Biotechnology and Food Microbiology, Wrocław University of Environmental and Life Sciences (Wrocław, Poland) as a glycerol stock (20% v/v) at –80 °C.

### Culture conditions

The modified MSM reported by Phulpoto et al.^26^ was prepared for biosurfactant production by *P. fluorescens* DR54. The MSM consisted of Na_2_HPO_4_ 2.2 g/L, MgSO_4_ × 7 H_2_O 0.6 g/L, FeSO_4_ × 7 H_2_O 0.01 g/L, NaCl 0.3 g/L, CaCl_2_ 0.02 g/L, and 0.1% trace elements solution containing ZnSO_4_ × 7 H_2_O 2.32 g/L, MnSO_4_ × 4 H_2_O 1.78 g/L, CuSO_4_ × 5 H_2_O 1.0 g/L, NH_4_MoO_4_ × 2 H_2_O 0.39 g/L, and KI 0.66 g/L. Different samples of glycerol and urea were added as carbon and nitrogen sources to this medium. Additionally, due to presence of three molecuels of leucine in the structure of the viscosinamide, this compound had an influence on the viscosinamide concentration. Furthermore, the addition of K_2_HPO_4_ to the production medium allowed for the maintenance of desired pH during fermentation and encouraged microbial growth Therefore, besides glycerol and urea, KH_2_PO_4_ and leucine were contained at different concentrations during the optimization process.

The growth of *P. fluorescens* DR54 with various samples of waste glycerol was tested in a microplate reader (Spark Cyto, Tecan, Switzerland) in MSM with 0.2% (w/v) of urea and 4% (w/v) glycerol (G1–G6). Growth profiles were analyzed in a 96-well plate containing 100 µL medium at 28 °C for 120 h with continuous shaking and analysis of OD_600_ every 30 min. The preinoculum was grown in Luria–Bertani (LB) medium for 24 h at 28 °C and 180 rpm. The experiments were performed in triplicate for each type of glycerol.

### Shake flask experiments

The preinoculum was grown in LB medium for 24 h in a 250 mL flask at 28 °C and 180 rpm. The shake flask experiments were cultivated in 300 mL baffled Erlenmeyer flasks containing 50 mL MSM supplemented with 0.2% (w/v) of urea and 4% (w/v) glycerol (G1–G6) at 28 °C, 180 rpm for 168 h on an incubation shaker (CERTOMAT IS; Sartorius Stedim Biotech, Aubagne, France). Each flask was inoculated with 1% of a preculture of *P. fluorescens* DR54. The experiment was performed in three biological replicates for each type of glycerol.

### Analytical methods

During shake flask experiments, 1.5 mL samples were collected every 24 h and were centrifuged (5 min, 5000 rpm). The concentration of glycerol was determined in the supernatants by high-performance LC (HPLC, UltiMate 3000; Thermo Fisher Scientific, London, UK) equipped with the HyperRez Carbohydrate H + Column (Thermo Fisher Scientific) and a refractive index detector (Shodex, Ogimachi, Japan). Trifluoroacetic acetic acid at a concentration 25 mM was applied as an elution agent. Elution was carried out at a flow rate of 0.6 mL/min at 65 °C. The concentration of viscosinamide was measured from cell-free supernatant using HPLC (Shimadzu, Kyoto, Japan) equipped with the Hypersil GOLD column (5 µm, 4.6 × 150 mm). As the mobile phase, solvents A (0.1% trifluoroacetic acid) and B (0.1% trifluoroacetic acid in acetonitrile) were applied in the following order: (% A:B v/v): 0 min (50:50), 5 min (20:80), 9 min (10:90), 15 min (0:100), 21 min (0:100), 24 min (50:50), and 25 min (50:50). Samples were injected in a 10 µL volume on the Hypersil GOLD column (5 µm, 4.6 × 150 mm) and eluted for 25 min. Elution was performed at a flow rate of 0.5 mL/min and detection was conducted at a 210 nm wavelength.

### Optimization of viscosinamide production

Viscosinamide production by *P. fluorescens* DR54 grown on glycerol was subjected to optimization using RSM, according to the Box–Behnken design. A non-linear regression model was built to determine the influence of the four input variables on the response (final viscosinamide concentration). The concentration of the following culture medium components served as independent variables: glycerol (X_1_), l-leucine (X_2_), monobasic potassium phosphate, anhydrous (X_3_), and urea (X_4_). Each independent variable was set at three experimental levels (− 1, 0, + 1), as listed in Table 5. The experimental layout comprised 27 runs (Table 5), including three replicates of the central point. Coefficients of the following polynomial equation were fitted to establish the regression model (2):

2 $${\text{Y }} = \, \beta_{0} + \, \beta_{{1}} {\text{X}}_{{1}} + \, \beta_{{2}} {\text{X}}_{{2}} + \, \beta_{{3}} {\text{X}}_{{3}} + \, \beta_{{4}} {\text{X}}_{{4}} + \, \beta_{{{11}}} {\text{X}}_{{1}} {\text{X}}_{{1}} + \, \beta_{{{22}}} {\text{X}}_{{2}} {\text{X}}_{{2}} + \beta_{{{33}}} {\text{X}}_{{3}} {\text{X}}_{{3}} + \, \beta_{{{44}}} {\text{X}}_{{4}} {\text{X}}_{{4}} + \, \beta_{{{12}}} {\text{X}}_{{1}} {\text{X}}_{{2}} + \, \beta_{{{13}}} {\text{X}}_{{1}} {\text{X}}_{{3}} + \, \beta_{{{14}}} {\text{X}}_{{1}} {\text{X}}_{{4}} + \, \beta_{{{23}}} {\text{X}}_{{2}} {\text{X}}_{{3}} + \, \beta_{{{24}}} {\text{X}}_{{2}} {\text{X}}_{{4}} + \, \beta_{{{34}}} {\text{X}}_{{3}} {\text{X}}_{{4}} ,$$

where Y is the predicted response; β_0_ is the intercept; β_1_, β_2_, β_3_, β_4_ are the linear regression coefficients; β_11_, β_22_, β_33_, β_44_ are the quadratic regression coefficients; and β_12_, β_13_, β_23_, β_24_, β_34_ are the interaction effects. Residual error was used in the tests of significance at p = 0.05. The design of the experiment, model analysis, and response optimization were performed using Statistica 13 software (TIBCO Software Inc., Palo Alto, CA, USA).

**Table 5:** Experimental layout of the Box–Behnken design with coded independent variables, obtained response outcomes, and predicted response values.

	Unit	Low (− 1)	Middle (0)	High (+ 1)		
X_1_: glycerol	g/L	10	55	100		
X_2_: leucine	g/L	0.1	2.55	5.0		
X_3_: phosphate	g/L	0.5	2.75	5.0		
X_4_: urea	g/L	1	5.5	10		

Run	Independent variables				Viscosinamide [g/L]	
	X_1_	X_2_	X_3_	X_4_	Actual response	Predicted response
1	− 1	− 1	0	0	0.847	− 0.181
2	1	− 1	0	0	0.613	1.979
3	− 1	1	0	0	2.330	1.003
4	1	1	0	0	0	1.066
5	0	0	− 1	− 1	1.539	1.858
6	0	0	1	− 1	0.271	− 0.702
7	0	0	− 1	1	0.729	1.742
8	0	0	1	1	6.483	6.203
9	0	0	0	0	4.709	6.425
10	− 1	0	0	− 1	0	1.493
11	1	0	0	− 1	0	− 1.455
12	− 1	0	0	1	0.765	0.827
13	1	0	0	1	8.886	5.999
14	0	− 1	− 1	0	3.204	2.826
15	0	1	− 1	0	0	− 0.725
16	0	− 1	1	0	0.759	0.091
17	0	1	1	0	4.925	3.911
18	0	0	0	0	5.318	6.425
19	− 1	0	− 1	0	0.076	− 0.316
20	1	0	− 1	0	1.676	1.839
21	− 1	0	1	0	0.486	1.678
22	1	0	1	0	0	1.746
23	0	− 1	0	− 1	0	− 0.015
24	0	1	0	− 1	0	0.631
25	0	− 1	0	1	3.167	3.891
26	0	1	0	1	2.144	3.514
27	0	0	0	0	9.247	6.425

Optimization studies allowed us to establish the best conditions for viscosinamide production. Hence, large-scale production in a 250 mL Erlenmeyer flask containing 50 mL of the medium, performed in the optimal conditions, i.e. glycerol 70.8 g/L, leucine 2.7 g/L, phosphate 3.7 g/L, and urea 9.3 g/L, was applied. The bacterial culture was inoculated with 1% of a pre-culture from *P. fluorescens* DSS73 grown for 24 h, and incubated at 28 °C and 180 rpm for 7 days. Finally, post-culture medium was centrifuged at 9500 g for 20 min to separate the precipitate. The concentration of viscosinamide was measured from cell-free supernatant using HPLC as described above.

### Viscosinamide purification

First, the post-culture medium was separated from bacterial cells during centrifugation (9500 rpm, 20 min). Additionally, the supernatant was filtered using a vacuum pump and then subjected to the lyophilization process using a Triad freeze-dryer by Labconco (Kansas City, Missouri, USA). Then the purification of viscosinamide was carried out using modified solid-phase extraction (SPE) reported by Alajlani et al.^56^. Crude biosurfactant was loaded onto cartridges of Chromabond C_18_ SPE (Macherey–Nagel, Düran, Germany) and washed with acetonitrile gradient (20%, 50%, 80%, and 100% acetonitrile–water (v/v)). Furthermore, the 80% and 100% acetonitrile–water (v/v) solutions including viscosinamide were concentrated using a rotary vacuum evaporator.

### Surface activity determination

The pendant drop method previously described by Hansen and Rødsrud^57^ was used to determine the ST of viscosinamide solutions with an optical contact angle (OCA) of 15 EC goniometer (DataPhysics Instruments GmbH, Filderstadt, Germany). Viscosinamide solutions of varying concentrations from 0 to 400 mg/L were prepared, and each solution was placed in a gastight syringe with a blunt tip needle (following the recommendation by Song and Springer^58^). To ensure a saturated vapor environment and minimize droplet evaporation, the needle tip was placed in a glass cuvette filled with a small amount of measured solution. Then the drop was suspended from the tip, and its images were captured every minute for 1 h. ST values were obtained using SCA20 software (DataPhysics Instruments GmbH), which performed a numerical fitting process by comparing the theoretical shape of a droplet predicted by the Young–Laplace equation with the recorded shape. ST for each concentration was determined three times, and the average values were reported in this study. The CMC value was determined from the ST versus viscosinamide concentration plot by finding the intersection point of two linear regression lines: the first of the linearly dependent region and the second passing through the plateau.

### Effect of viscosinamide on lipase activity

Lipase from CRL was purchased from Sigma-Aldrich Chemical Co. (St. Louis, MO, USA). The enzymatic activity was determined as reported previously by Janek et al.^47^ with modifications given below. To determine the effect of lipopeptide biosurfactant on CRL activity, six concentrations (5, 10, 20, 40, 80, 160 mg/L) of viscosinamide were used. All tests were performed in comparison with control. In these experiments, the enzyme was pre-incubated with viscosinamide for 60 min at 25 °C, and the residual activity was tested using p-nitrophenyl octanoate (pNPO, C8) as the substrate. The reaction contained 1 mL substrate solution containing 0.5 mM pNPO (dissolved in acetonitrile at first) in 50 mM Tris–HCl buffer (pH 7.4) and 40 µL of appropriately diluted enzyme (corresponding to a final concentration of 0.1 mg/mL protein in the reaction mixture). The reactions were incubated at 25 °C for 10 min. One unit of CRL activity was defined as the amount of enzyme liberating 1 μmol p-nitrophenol (pNP) per minute. All measurements were performed in three independent experiments.

### CD measurement

CD spectra were recorded as previously described by Janek et al.^52^ on the J-1500 Circular Dichroism Spectrophotometer (JASCO Co., Tokyo, Japan) at 25 °C under a constant flow of nitrogen gas. The CD spectra were measured in the range of 200–260 nm. Each spectrum was the average of nine successive scans. The scanning speed was set at 50 nm per minute. The scans of 50 mM Tris–HCl buffer solution (pH 7.4) used as a blank were recorded under the same conditions and subtracted from the experimental spectra. The analysis of secondary structural contents was performed using the Jasco spectropolarimeter software (JWSSE-513 protein secondary structural analysis program).

### Supplementary Information

Supplementary Tables.

## Data Availability

The datasets generated during and/or analysed during the current study are available from the corresponding author on reasonable request tomasz.janek@upwr.edu.pl.

## References

[1] Das P, Mukherjee S, Sen R (2009). Substrate dependent production of extracellular biosurfactant by a marine bacterium. Bioresour. Technol..

[2] Santos DKF, Rufino RD, Luna JM, Santos VA, Sarubbo LA (2016). Biosurfactants: Multifunctional biomolecules of the 21st century. Int. J. Mol. Sci..

[3] Domínguez Rivera Á, Martínez Urbina MÁ, López y López VE (2019). Advances on research in the use of agro-industrial waste in biosurfactant production. World J. Microbiol. Biotechnol..

[4] Deepika KV, Kalam S, Ramu Sridhar P, Podile AR, Bramhachari PV (2016). Optimization of rhamnolipid biosurfactant production by mangrove sediment bacterium *Pseudomonas*
*aeruginosa* KVD-HR42 using response surface methodology. Biocatal. Agric. Biotechnol..

[5] Seghal Kiran G, Anto Thomas T, Selvin J, Sabarathnam B, Lipton AP (2010). Optimization and characterization of a new lipopeptide biosurfactant produced by marine *Brevibacterium*
*aureum* MSA13 in solid state culture. Bioresour. Technol..

[6] Kumar A (2021). Microbial biosurfactant: A new frontier for sustainable agriculture and pharmaceutical industries. Antioxidants.

[7] Roy A (2018). A review on the biosurfactants: Properties, types and its applications. J. Fundam. Renew. Energy Appl..

[8] Moldes AB (2021). Synthetic and bio-derived surfactants versus microbial biosurfactants in the cosmetic industry: An overview. Int. J. Mol. Sci..

[9] Karlapudi AP (2020). Evaluation of anti-cancer, anti-microbial and anti-biofilm potential of biosurfactant extracted from an Acinetobacter M6 strain. J. King Saud Univ. Sci..

[10] Sen S, Borah SN, Bora A, Deka S (2017). Production, characterization, and antifungal activity of a biosurfactant produced by *Rhodotorula*
*babjevae* YS3. Microb. Cell Fact..

[11] Satpute SK, Mone NS, Das P, Banat IM, Banpurkar AG (2019). Inhibition of pathogenic bacterial biofilms on PDMS based implants by *L*. *acidophilus* derived biosurfactant. BMC Microbiol..

[12] Oni FE (2020). Biosynthesis and antimicrobial activity of pseudodesmin and viscosinamide cyclic lipopeptides produced by pseudomonads associated with the cocoyam rhizosphere. Microorganisms.

[13] Nielsen TH, Sørensen J (2003). Production of cyclic lipopeptides by *Pseudomonas*
*fluorescens* strains in bulk soil and in the sugar beet rhizosphere. Appl. Environ. Microbiol..

[14] Carolin CF, Kumar PS, Ngueagni PT (2021). A review on new aspects of lipopeptide biosurfactant: Types, production, properties and its application in the bioremediation process. J. Hazard Mater..

[15] Zanotto AW, Valério A, de Andrade CJ, Pastore GM (2019). New sustainable alternatives to reduce the production costs for surfactin 50 years after the discovery. Appl. Microbiol. Biotechnol..

[16] Janek T (2021). Sustainable surfactin production by *Bacillus*
*subtilis* using crude glycerol from different wastes. Molecules.

[17] Janek T, Mirończuk AM, Rymowicz W, Dobrowolski A (2020). High-yield expression of extracellular lipase from *Yarrowia*
*lipolytica* and its interactions with lipopeptide biosurfactants: A biophysical approach. Arch. Biochem. Biophys..

[18] Ozyilmaz E, Eski F (2020). Effect of cyclic and acyclic surfactants on the activity of *Candida*
*rugosa* lipase. Bioprocess Biosyst. Eng..

[19] Rubingh DN (1996). The influence of surfactants on enzyme activity. Curr. Opin. Colloid Interface Sci..

[20] Holmberg K (2018). Interactions between surfactants and hydrolytic enzymes. Colloids Surf. B Biointerfaces.

[21] Melani NB, Tambourgi EB, Silveira E (2020). Lipases: From production to applications. Sep. Purif. Rev..

[22] Sarmah N (2018). Recent advances on sources and industrial applications of lipases. Biotechnol. Prog..

[23] Chandra P, Enespa, Singh R, Arora PK (2020). Microbial lipases and their industrial applications: A comprehensive review. Microb. Cell Fact..

[24] Mandenius CF, Brundin A (2008). Bioprocess optimization using design-of-experiments methodology. Biotechnol. Prog..

[25] Gilman J, Walls L, Bandiera L, Menolascina F (2021). Statistical design of experiments for synthetic biology. ACS Synth. Biol..

[26] Phulpoto IA (2021). Bioprospecting of rhamnolipids production and optimization by an oil-degrading *Pseudomonas* sp. S2WE isolated from freshwater lake. Bioresour. Technol..

[27] Kumar AP, Janardhan A, Radha S, Viswanath B, Narasimha G (2015). Statistical approach to optimize production of biosurfactant by *Pseudomonas*
*aeruginosa* 2297. 3 Biotech.

[28] de Sousa M (2014). Crude glycerol from biodiesel industry as substrate for biosurfactant production by *Bacillus*
*subtilis* ATCC 6633. Braz. Arch. Biol. Technol..

[29] Biniarz P, Coutte F, Gancel F, Łukaszewicz M (2018). High-throughput optimization of medium components and culture conditions for the efficient production of a lipopeptide pseudofactin by *Pseudomonas*
*fluorescens* BD5. Microb. Cell Fact..

[30] Liu S, Tang MH, Cheng JS (2022). Fermentation optimization of surfactin production of *Bacillus*
*amyloliquefaciens* HM618. Biotechnol. Appl. Biochem..

[31] Liu JF, Yang J, Yang SZ, Ye RQ, Mu BZ (2012). Effects of different amino acids in culture media on surfactin variants produced by *Bacillus*
*subtilis* TD7. Appl. Biochem. Biotechnol..

[32] Ma M, Li R, Du Y, Tang Z, Zhou W (2013). Analysis of antibacterial properties of naturally colored cottons. Text. Res. J..

[33] Lotfy WA, Hassan SWM, Abd El-Aal AA, Ghanem KM (2019). Enhanced production of di-(2-ethylhexyl) phthalate (DEHP) by *Bacillus*
*subtilis* AD35 using response surface methodology (RSM). Biotechnol. Biotechnol. Equip..

[34] Patil SA, Surwase SN, Jadhav SB, Jadhav JP (2013). Optimization of medium using response surface methodology for l-DOPA production by *Pseudomonas* sp. SSA. Biochem. Eng. J..

[35] Singh SP, Bharali P, Konwar BK (2013). Optimization of nutrient requirements and culture conditions for the production of rhamnolipid from *Pseudomonas*
*aeruginosa* (MTCC 7815) using Mesua ferrea seed oil. Indian J. Microbiol..

[36] Clarke KG, Ballot F, Reid SJ (2010). Enhanced rhamnolipid production by *Pseudomonas*
*aeruginosa* under phosphate limitation. World J. Microbiol. Biotechnol..

[37] Ghribi D, Ellouze-Chaabouni S (2011). Enhancement of *Bacillus*
*subtilis* lipopeptide biosurfactants production through optimization of medium composition and adequate control of aeration. Biotechnol. Res. Int..

[38] Baskaran SM (2021). Valorization of biodiesel side stream waste glycerol for rhamnolipids production by *Pseudomonas*
*aeruginosa* RS6. Environ. Pollut..

[39] Hentati D (2019). Production, characterization and biotechnological potential of lipopeptide biosurfactants from a novel marine *Bacillus*
*stratosphericus* strain FLU5. Ecotoxicol. Environ. Saf..

[40] Mouafo TH, Mbawala A, Ndjouenkeu R (2018). Effect of different carbon sources on biosurfactants’ production by three strains of *Lactobacillus* spp. Biomed. Res. Int..

[41] Saini HS (2008). Efficient purification of the biosurfactant viscosin from *Pseudomonas*
*libanensis* strain M9–3 and its physicochemical and biological properties. J. Nat. Prod..

[42] Renard P (2019). Cloud microorganisms, an interesting source of biosurfactants. Surfactants Deterg..

[43] Neu TR, Hfirtner T, Poralla K (1990). Applied microbiology biotechnology surface active properties of viscosin: A peptidolipid antibiotic*. Appl. Microbiol. Biotechnol..

[44] Hildebrand PD, Braun PG, McRae KB, Lu X (1998). Role of the biosurfactant viscosin in broccoli head rot caused by a pectolytic strain of *Pseudomonas*
*fluorescens*. Can. J. Plant Pathol..

[45] Grochulski P, Li Y, Schrag JD, Cygler M (1994). Two conformational states of *Candida*
*rugosa* lipase. Protein Sci..

[46] Zhang R (2018). Interaction of a digestive protease, *Candida*
*rugosa* lipase, with three surfactants investigated by spectroscopy, molecular docking and enzyme activity assay. Sci. Total Environ..

[47] Janek T, Mirończuk AM, Rymowicz W, Dobrowolski A (2020). High-yield expression of extracellular lipase from Yarrowia lipolytica and its interactions with lipopeptide biosurfactants: A biophysical approach. Arch. Biochem. Biophys..

[48] Kelly SM, Jess TJ, Price NC (2005). How to study proteins by circular dichroism. Biochim. Biophys. Acta Proteins Proteom..

[49] Zsila F (2015). Apparent circular dichroism signature of stirring-oriented DNA and drug-DNA complexes. Int. J. Biol. Macromol..

[50] Barrow CJ, Yasuda A, Kenny PTM, Zagorski M (1992). Solution conformations and aggregational properties of synthetic amyloid b-peptides of Alzheimer’s disease. J. Mol. Biol..

[51] Fan L, Xie P, Wang Y, Huang Z, Zhou J (2018). Biosurfactant-protein interaction: Influences of mannosylerythritol lipids-A on β-glucosidase. J. Agric. Food Chem..

[52] Janek T, Czyżnikowska Ż, Łukaszewicz M, Gałęzowska J (2019). The effect of Pseudomonas fluorescens biosurfactant pseudofactin II on the conformational changes of bovine serum albumin: Pharmaceutical and biomedical applications. J. Mol. Liq..

[53] Gull N (2009). Spectroscopic studies on the interaction of cationic surfactants with bovine serum albumin. Colloids Surf. B Biointerfaces.

[54] Dobrowolski A, Mituła P, Rymowicz W, Mirończuk AM (2016). Efficient conversion of crude glycerol from various industrial wastes into single cell oil by yeast Yarrowia lipolytica. Bioresour. Technol..

[55] Normander B, Hendriksen NB, Nybroe O (1999). Green fluorescent protein-marked pseudomonas fluorescens: Localization, viability, and activity in the natural barley rhizosphere. Appl. Environ. Microbiol..

[56] Alajlani M, Shiekh A, Hasnain S, Brantner A (2016). Purification of bioactive lipopeptides produced by *Bacillus*
*subtilis* strain BIA. Chromatographia.

[57] Hansen FK, Rødsrud G (1991). Surface tension by pendant drop. I. A fast standard instrument using computer image analysis. J. Colloid Interface Sci..

[58] Song B, Springer J (1996). Determination of interfacial tension from the profile of a pendant drop using computer-aided image processing. J. Colloid Interface Sci..

