# Supplementary material for: Bioconversion of waste glycerol into viscosinamide by Pseudomonas fluorescens DR54 and its activity evaluation
Source: Sci Rep. 2024 Jan 17;14:1531. doi: 10.1038/s41598-024-51179-4 (PMC10794706; doi:10.1038/s41598-024-51179-4)
Supplement: Supplementary file 1 — Supplementary Tables. [file 41598_2024_51179_MOESM1_ESM.docx]

| Symbol | G1 | G2 | G3 | G4 | G5 | G6 |
| --- | --- | --- | --- | --- | --- | --- |
| Waste product derived from [%]: | Pure (the control) | Stearin production | Bio-diesel | Bio-diesel | Soap production | Bio-diesel |
| Glycerol content | 100 | 42 | 80 | 50 | 80 | 87 |
| Nitrogen content | 0 | 0.136 | 0.014 | 0.078 | 0.041 | 0.023 |
| NaCl | 0 | 1.23 | 5.47 | 3.04 | 7.59 | 0.20 |
| Ash | 0 | 1.35 | 6.34 | 3.62 | 8.76 | 0.93 |
| Water | 0 | 55.3 | 8.16 | 43.42 | 3.6 | 11.85 |

**Table S1** Percentage of glycerol, nitrogen and other compounds in the batches of crude glycerol.

**Table S2** The content of elements in the various batches of crude glycerol.

| Crude  glycerol | The content of elements (mg/kg) | | | | | | | |
| --- | --- | --- | --- | --- | --- | --- | --- | --- |
|  | Cu | Mg | Fe | Zn | K | Na | Cl | Ca |
| G2 | 0.03 ± 0.01 | 5.305 ± 1.06 | 458.38 ± 45.84 | 1.251 ± 0.25 | 63.137 ± 6.31 | 5224.84 ± 261.24 | 7400 ± 59.26 | 461.95 ± 46.19 |
| G3 | 0.39 ± 0.04 | 22.55 ± 1.02 | 5.91 ± 0.20 | 1.41 ± 0.13 | 65.89 ± 4.88 | 23120.03 ± 764.10 | 33200 ± 425.56 | 132.21 ± 5.45 |
| G4 | 0.12 ± 0.08 | 15.26 ± 0.45 | 2.31 ± 0.68 | 1.13 ± 0.34 | 72.74 ± 8.98 | 13102.66 ± 519.93 | 18500 ± 30.00 | 97.06 ± 9.44 |
| G5 | 0.66 ± 0.08 | 5.454 ± 0.44 | 26.42 ± 1.20 | 1.30 ± 0.40 | 231.32 ± 4.77 | 31632.89 ± 521.66 | 46000 ± 21.20 | 52.36 ± 10.93 |
